# COVID-19 pandemic-related drugs and microplastics from mask fibers jointly affect soil functions and processes

**DOI:** 10.1007/s11356-024-34587-x

**Published:** 2024-08-05

**Authors:** Jeane dela Cruz, Daniel Lammel, Shin Woong Kim, Mohan Bi, Matthias Rillig

**Affiliations:** 1 Institute of Biology, Freie Universität Berlin, 14195 Berlin, Germany; 2 Berlin-Brandenburg Institute of Advanced Biodiversity Research, 14195 Berlin, Germany

**Keywords:** Pharmaceutical products, FFP2 mask, Global change factors, Microbial activity, Environmental side effects, Soil pollution

## Abstract

The COVID-19 pandemic has led to an unprecedented increase in pharmaceutical drug consumption and plastic waste disposal from personal protective equipment. Most drugs consumed during the COVID-19 pandemic were used to treat other human and animal diseases. Hence, their nearly ubiquitous presence in the soil and the sharp increase in the last 3 years led us to investigate their potential impact on the environment. Similarly, the compulsory use of face masks has led to an enormous amount of plastic waste. Our study aims to investigate the combined effects of COVID-19 drugs and microplastics from FFP2 face masks on important soil processes using soil microcosm experiments. We used three null models (additive, multiplicative, and dominative models) to indicate potential interactions among different pharmaceutical drugs and mask MP. We found that the multiple-factor treatments tend to affect soil respiration and FDA hydrolysis more strongly than the individual treatments. We also found that mask microplastics when combined with pharmaceuticals caused greater negative effects on soil. Additionally, null model predictions show that combinations of high concentrations of pharmaceuticals and mask MP have antagonistic interactions on soil enzyme activities, while the joint effects of low concentrations of pharmaceuticals (with or without MP) on soil enzyme activities are mostly explained by null model predictions. Our study underscores the need for more attention on the environmental side effects of pharmaceutical contamination and their potential interactions with other anthropogenic global change factors.

**Supplementary Information:**

The online version contains supplementary material available at 10.1007/s11356-024-34587-x.

## Introduction

On March 11, 2020, the World Health Organization declared Coronavirus disease 2019 (COVID-19) a pandemic (WHO 2020a). As of October 2023, more than 700 million cases have been confirmed and over 6 million deaths have been reported globally (WHO 2023a). Because of the rapid number of cases and large number of deaths, medical doctors and patients in many countries chose to use “repurposed” drugs developed for other diseases hoping to prevent or cure the severe acute respiratory syndrome coronavirus 2 (SARS-CoV-2). Despite unknown efficacy of the treatments, there was a large global increase in the sale of medicines such as azithromycin, ivermectin, and hydroxychloroquine (Del Fiol et al. 2022; Schaffer et al. 2022; Urquhart 2023). Given the enormous number of individuals needing medication, this unprecedented public health threat has resulted in large-scale consumption of pharmaceutical drugs in the last 3 years (Gonzalez-Zorn 2021; Nandi et al. 2023). This was also evident in the increased detection of these compounds in different environmental water matrices (Domingo-Echaburu et al. 2022; Galani et al. 2021).

Most of the drugs of choice for COVID-19 treatment have long existed and have been used to treat other diseases of both humans and animals. Ivermectin, for example, is one of the most widely used drugs to control human and animal parasites (Laing et al. 2017). Its use was also proposed for mass drug administration (MDA) against malaria in highly endemic regions (Chaccour and Rabinovich 2019; Omura and Crump 2017) as resistance to chloroquine is increasing (Badhan et al. 2018). Later, it was found to inhibit the replication of the SARS-CoV-2 virus (Caly et al. 2020). Likewise, azithromycin, a broad-spectrum antibiotic, is used to treat several illnesses including skin and respiratory infections, and is often used for the mass treatment of trachoma (Alasmar et al. 2022; WHO 2023b). Azithromycin was the second most prescribed antibiotic for outpatients in the USA in 2022 (CDC 2023a). Antivirals could be among the drugs with a significant increase in consumption during the pandemic (Gold et al. 2022). Remdesivir was the first antiviral drug clinically proven effective against the virus (Beigel et al. 2020) and has since been used alongside other antivirals to treat COVID-19 patients (CDC 2023b). While it is imperative to combat the pressing health issues associated with the pandemic, including antimicrobial resistance, it is also important to scrutinize other non-target effects of massive pharmaceutical drug use. After all, abrupt increases in pharmaceutical usage in pandemic scenarios will undeniably discharge enormous amounts of these compounds into the environment.

Pharmaceutical drugs are not fully metabolized in the body. For example, up to 50–60% of antiparasitic and antiviral drugs are reported to be discharged via urine and feces in their active form or as metabolites and become part of the influents that enter sewage treatment facilities (Jjemba 2006; Kuroda et al. 2021). However, these facilities cannot eliminate pharmaceutical products and by-products (Morales-Paredes et al. 2022; Nippes et al. 2021). They are frequently detected in effluents suggesting persistence through the treatment process (Tran et al. 2018). Effluents and sludge are then disposed of in the environment where they reach surface water and soil. Agricultural soils are contaminated by these chemical-laden discharges when treated water is re-used for irrigation and biosolids are applied as fertilizers which may persist for a longer period (Borgman and Chefetz 2013; Gottschall et al. 2012; Gravesen and Judy 2020). However, they may reach the environment untreated, such as via accidental and improper disposal of household wastes. As several of these products are also used in veterinary medicine, consequent release via animal excreta and organic fertilizer application from animal manure increased the pharmaceutical residue in the soil (Kaczala and Blum 2016; Wohde et al. 2016). With removal efficiencies exhibiting high variability, gradual accumulation in the environment is possible from concurrent, excessive, and continuous discharge (Bayati et al. 2021; Morales-Paredes et al. 2022; Sui et al. 2015; Tran et al. 2018). In addition to chronic application, many of these compounds remain relatively persistent after discharge. Consequently, elevated levels due to accumulation may potentially result in contamination hotspots posing a greater risk to the environment (Löffler et al. 2005; Walters et al. 2010). Since 2009, azithromycin has been included on the growing list of contaminants of emerging concern by the US Environmental Protection Agency (EPA 2009) and by the European Union via Decision 495/2015 (European Commission 2015; Sousa et al. 2019).

During the pandemic, the WHO employed different measures to prevent the spread of the virus. Besides early detection and treatment, the use of personal protective equipment (PPE) like face masks was also practiced. In early 2020 when mask use was mostly limited to medical personnel, WHO estimated around 89 million medical masks are required monthly (WHO 2020b). As the pandemic progressed, mask use became a universal requirement. Although the use of face masks effectively reduced COVID-19 cases (Leech et al. 2022; Mitze et al. 2020), this also led to a large amount of plastic waste being discharged (Shukla et al. 2022; F. Wang et al. 2022). Consequently, mask microplastics (mask MP, plastic particles < 5 mm) generated from the physico-chemical degradation of face masks eventually add to the contamination burden in terrestrial and aquatic environments (Fadare and Okoffo 2020; Jiang et al. 2023; Morgana et al. 2021). Protective masks are made from different plastic materials like polypropylene, polyurethane, polyacrylonitrile, polystyrene, polycarbonate, polyethylene, or polyester (Aragaw 2020). Similar to pharmaceutical products, one way microplastics (MP) can enter agricultural fields is via the application of contaminated water for irrigation and sewage sludge as soil amendments (Bläsing and Amelung 2018; Edo et al. 2020). High amounts of plastic particles were found to accumulate in agricultural soil following these agriculture management practices (Huang et al. 2023; G. S. Zhang and Liu 2018). A range of studies have shown the impacts of microplastics on soil properties and functions from being negative (De Souza Machado et al. 2018; Lozano et al. 2021a, 2021b; Zhao et al. 2021) to slightly positive (Lozano et al. 2021a, 2021b).

While pharmaceutical drugs are gaining research interest and are now considered emerging environmental contaminants (Osuoha et al. 2023), studies were mostly focused on aquatic environments (Ebele et al. 2017; Ortiz de García et al. 2014; Richmond et al. 2017; Sui et al. 2015). Likewise, reports on the environmental impact of COVID-19 pandemic–related pharmaceutical products are centered on the detection of the compounds in aquatic systems and wastewater treatment plants (WWTP) and the effect on aquatic organisms (Domingo-Echaburu et al. 2022; Gwenzi et al. 2022; Kumari and Kumar 2022; Kuroda et al. 2021; Nippes et al. 2021; Pashaei et al. 2022).

In this study, we focused on the impacts of different classes of pharmaceutical drugs mainly used during the pandemic and their effects on soil properties, functions, and microbial diversity. Furthermore, as they exist as a cocktail of compounds in nature, we investigated their combined effects and their interaction with other organic contaminants such as microplastics. We used a soil microcosm experiment to test (1) how single-drug treatments differ in their effects on soil functions and properties compared to when applied in combination and (2) whether the addition of microplastics derived from FFP2 mask in the combination will elicit a different response. We hypothesize that pharmaceutical drugs and mask MP will have distinct effects on soil parameters due to the difference in their physicochemical properties and mechanisms of action. We expect a more drastic impact from the multiple-factor treatments. Additionally, we hypothesized different pharmaceutical drugs and mask MP may have synergistic or antagonistic interactions when they are jointly applied. To the best of our knowledge, we provide here first evidence of the impacts of COVID-19 pandemic–related drugs and mask MP and how their combination can alter soil functions.

## Materials and methods

### Pharmaceutical compounds and mask microplastics

The chemical structures and physicochemical properties of remdesivir (antiviral), azithromycin (antibacterial), and ivermectin (antiparasitic) are presented in Table S2. To prepare the stock solutions, the compounds were initially dissolved in DMSO and subsequently added with deionized water. From these, working solutions of low and high concentrations were prepared based on available maximum reported concentration (mrc) or maximum Environmental Concentration (MEC) data for soil (Table S2). The microplastics from FFP2 face mask (Virshields© Filtering Half Mask VS005 FFP2 NR, Wroclaw, Poland) were isolated from the inner layer. This layer was identified by the manufacturer as polypropylene. These were manually cut into smaller pieces using scissors. Further, a kitchen mill (Rommelsbacher, Germany) was used to prepare even smaller and more homogeneous particles. The MP was briefly microwaved (3 min, 500 W) to reduce microbial populations (De Souza Machado et al. 2018).

### Experimental design

The experiment was designed with three levels of combination treatments (0, 1, 3, and 4): three different pharmaceutical compounds (remdesivir, ivermectin, azithromycin) and one microplastic (polypropylene from FFP2 mask) (Table S1). Single-factor treatments involved individual pharmaceutical compounds and mask MP. Three-factor treatments included all different combinations of compounds and mask MP with either one compound or microplastic excluded from the combination resulting in four different combinations. The four-factor treatment included all pharmaceutical compounds and the mask MP. In addition, each treatment, whether individual or in combination, included a low and a high concentration. These pollutants belonging to different classes are expected to deliver different effects on soil and microbial communities due to their varying mechanisms of action (Table S2). They were chosen due to their long history of usage and were among the common drugs of choice for COVID-19 treatment. Hence, increased detection in different matrices were reported (Chacca et al. 2022; Morales-Paredes et al. 2022).

The experimental units were prepared using 50 ml conical tubes (Corning Inc., Corning, USA) filled with 40 g of soil. The tubes had four vents in the cap allowing gas exchange but layered with a hydrophobic membrane to avoid contamination. First, the soil (Albic Luvisol) collected from the agricultural field station of Freie Universität Berlin (52° 28′ 00.9′′ N 13° 17′ 53.8′′ E) was air-dried, sieved (a 2-mm mesh size), homogenized, and stored at 4 °C. A previously sterilized loading soil (autoclaved, 121 °C for 1 h) of about 5 g was supplemented with the corresponding pharmaceutical compounds and was allowed to evaporate. This loading soil was used to avoid exaggerated effects of chemicals and allow effective mixing into the soil system. Then, 0.12 g of microplastic (0.4% w/w) was added to the remaining 35 g of soil and mixed manually. The microplastic concentration was determined as the upper limit concentration at which soil experienced minimal changes in volume (De Souza Machado et al. 2018). The loading soil was then thoroughly mixed with the 35 g soil for 3 min. Microcosms were randomly placed in an incubator set at 25 °C for 6 weeks. Soil moisture was maintained at 60% water-holding capacity. All treatments were replicated eight times. Ten additional tubes were included as the control group without any pharmaceutical product or microplastics. Control tubes were mixed in the same manner as the treatment group to receive the same amount of disturbance.

### Measurement of response variables

The following response variables were measured: physical properties (pH, water-stable soil aggregates), microbial activity (soil respiration, FDA hydrolysis), microbial abundance (bacteria and fungi), and nutrient cycling (litter decomposition and enzyme activities). Soil respiration was measured as CO_2_ concentration (ppm h^−1^ g^−1^ soil) using an infrared gas analyzer (LI-6400XT, LI-COR Inc., Bad Homburg, Germany) at different time points (days 5, 28, and 42). All other response variables were measured at the end of the incubation period. Soil pH was measured with 0.01 M CaCl_2_ solution using a pH meter (Knick, Germany). Water-stable soil aggregates were measured using a wet sieving apparatus (Eijkelkamp, Giesbeek, the Netherlands) following an established protocol (Kemper and Rosenau 1986; Liang et al. 2019). Litter decomposition was determined using prepared nylon bags filled with green tea leaves (Lipton Green Tea Sencha, Japan). The loss in the litter dry weight during the incubation was used to calculate the decomposition rate. Four enzymes essential for nutrient cycling were measured namely, β-glucosidase (cellulose degradation), β-D-cellobiosidase (cellulose degradation), N-acetyl-β-glucosaminidase (chitin degradation), and phosphatase (organic phosphorus mineralization) using a high-throughput microplate assay. Likewise, fluorescein diacetate hydrolase (FDA) was measured to represent general soil microbial activity. Microbial abundance was also measured using quantitative polymerase chain reactions (qPCR) with CFX 96 Real-Time System (BioRad Laboratory, Hercules, USA). First, soil DNA was extracted using DNeasy PowerSoil Pro Kit (Qiagen GmbH, Germany) following the manufacturer’s instructions. Bacterial DNA was amplified using the universal primers 515F (5′-GTGCCAGCMGCCGCGGTAA-3′) and 806R (5′-GGACTACHVGGGTWTCTAAT-3′) (Xu et al. 2022), while for fungal DNA, fITS7 (5′‐ GTGARTCATCGAATCTTTG‐3′) and the ITS4 primers (5′‐TCCTCCGCTTATTGATATGC‐3′) were used (Ihrmark et al. 2012). A more detailed procedure is available in the Supplementary Information.

### Statistical analysis

All data analysis and visualization were done in R (v4.3.1; R Core Team 2023). The effect sizes and 95% confidence intervals (CIs) of single and multiple factor treatments were estimated by a bootstrap method with 10,000 permutations. Plots that visualize the effect sizes and distributions of raw data of every treatment group were generated. Positive effect means the measured response variable was higher in the treatment compared to the control. The negative effect indicates the opposite. Generalized linear model (glm) followed by a multiple comparison test using the Dunnett’s test with the glht() function in the R package “multcomp” (Hothorn et al. 2008) were used to simultaneously compare each treatment with the control (Dunnett 1955). Model residuals were checked for normality and heteroscedasticity. The relationships among the different response variables were analyzed using principal component analysis and presented as biplot. The prcomp() function in the basic “stats'' package was used for this purpose. To test the potential interactions of multiple-factor treatments on soil properties, three null model assumptions (additive, multiplicative, and dominative) were employed (Schäfer and Pigott 2018) to estimate the joint effects of multiple factor treatments based on their component factors’ effect sizes. In the additive assumption, joint factor effects are estimated by adding up all component factor effect sizes. In the dominative case, the joint effect of multiple factors is equal to the overriding factor with largest absolute effect size. In the multiplicative assumption, combined effects are calculated by multiplying the proportional changes of single-factor effects on control. All null models assume that there is no interaction among factors. The interactions among factors are detected based on the deviation of actual data from the null model predictions. If the actual joint effect is significantly different from any of the three null model predictions, we consider that there is synergistic or antagonistic interaction among the component factors. Plots were generated with the ggplot2 package (Wickham 2016).

## Results

### Effects on microbial activity (soil respiration and FDA hydrolysis)

**Fig. 1:**
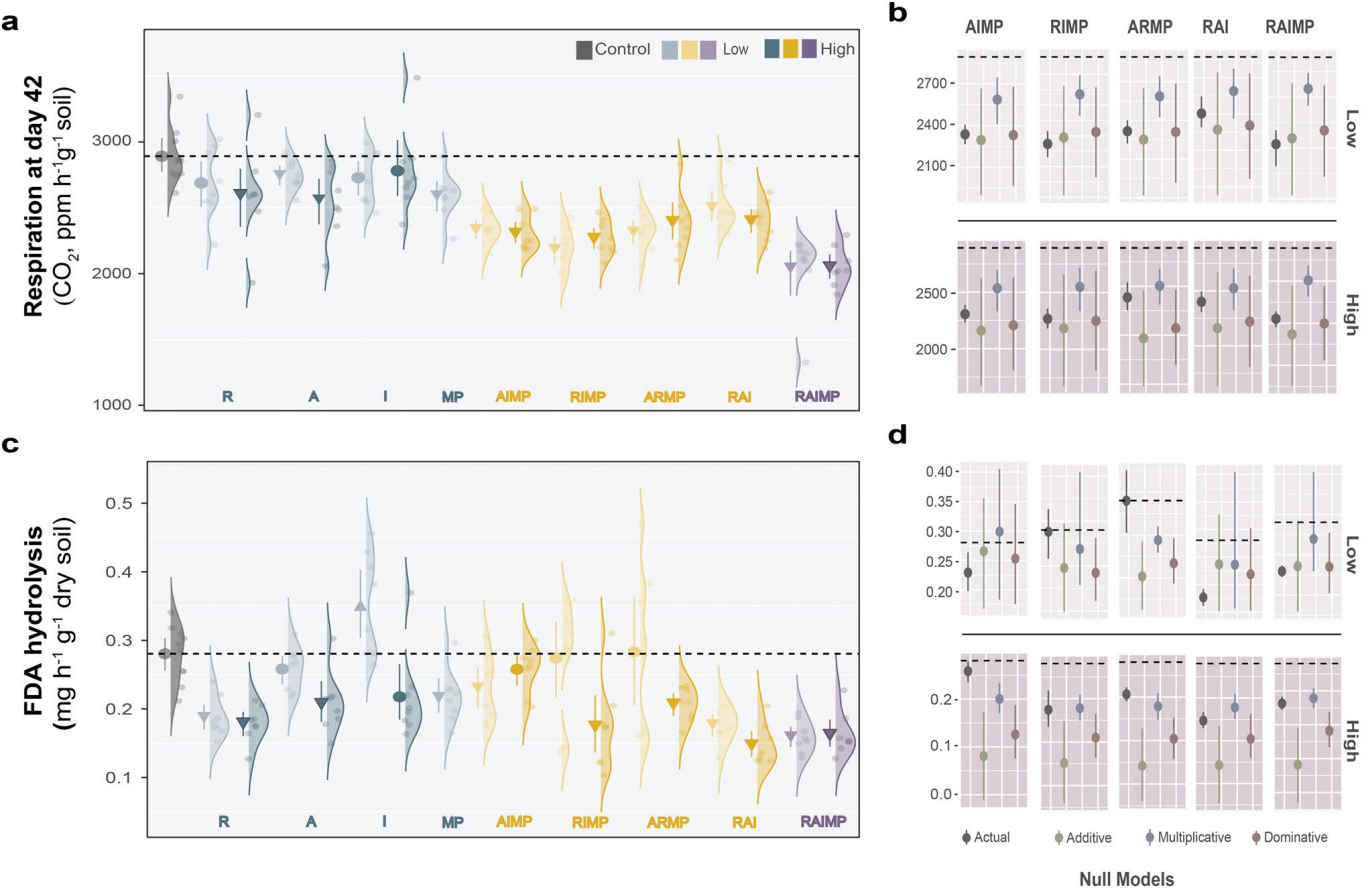
Effects of the individual treatment (remdesivir, R; azithromycin, A; ivermectin, I; mask microplastic, MP) and combinations of pharmaceutical drugs and microplastics on soil respiration measured on day 42 (**a**) and FDA hydrolysis (**c**). Density plots (**a** and **c**) display the data distributions with raw data shown as dots. Unpaired mean (effect magnitude) is presented as circles or arrows with corresponding 95% confidence intervals (effect precision) presented as vertical lines. Negative and positive effects are presented as arrows pointing downwards and upward, respectively while neutral effects are presented as circles. Lighter hue indicates low concentration and darker hue indicates high concentration. Null models were used to predict the impacts of multiple-factor treatments on soil processes using individual treatment effects (**b** and **d**). Error bars of multiple factor interactions in the null model plots were generated by boot-strapped values with 1000 iterations. Null models for low-concentration and high-concentration treatments are presented in the upper and lower panels, respectively. Factor levels are displayed in different colors:  - control;  - single-factor;  -three-factor; and  - four-factor treatments

We measured soil respiration at different time points (days 5, 28, and 42). Day 5 showed the highest respiration rates, while days 28 and 42 were substantially lower. On days 5 and 28 (Figure S1A and C, respectively), significant reductions were observed under the multiple-factor treatments while single-factor treatments had neutral effects. On day 42 (Fig. 1a), soil respiration was inhibited in both the single-factor and the multiple-factor treatments. In all measurements, we found no difference in response between low and high concentrations of chemical pollutants. There were no distinct differences in null model assumptions (additive, multiplicative, and dominative) **(**Fig. 1b). We also evaluated overall microbial activity by FDA hydrolysis (Fig. 1c). We found an overall decrease regardless of whether the compounds were added individually or in combination. Excluding mask MP (RAI) in the combination did not improve the effect. There were significant differences between the actual data and null model assumptions (ARMP-low and AIMP-high), and the FDA activity of these treatments was higher than the predictions (Fig. 1d). Despite the changes observed in these proxies for soil microbial activities, litter decomposition was marginally affected, only showing a slightly higher decomposition rate in the treated soil compared to the control (Figure S4A).

### Effects on soil processes

**Fig. 2:**
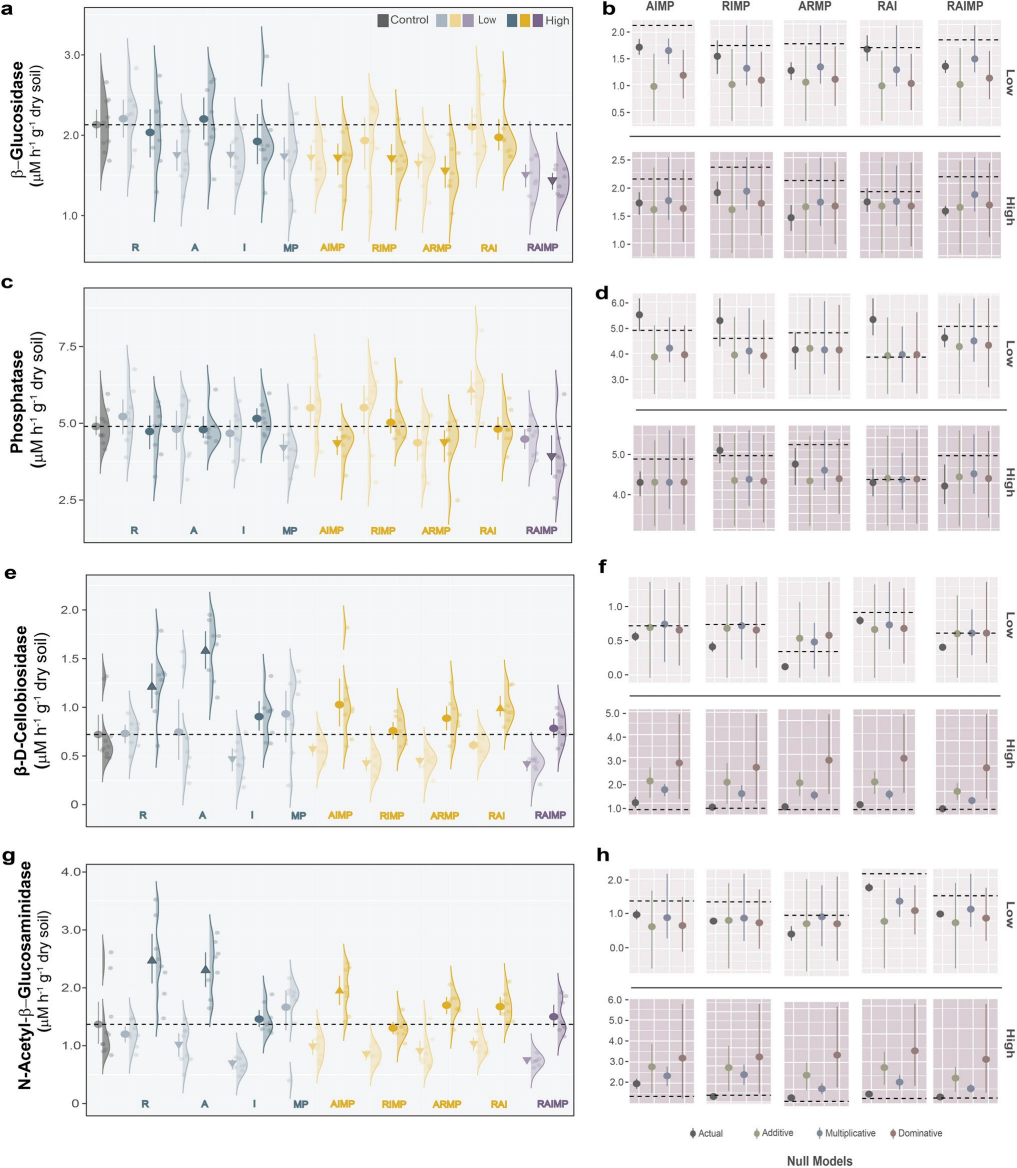
Effects of the individual treatment (remdesivir, R; azithromycin, A; ivermectin, I; mask microplastic, MP) and combinations of pharmaceutical drugs and microplastics on soil enzymes (**a**, **b**, **c**, **d**, **e**, **f**, **g**, **h**). Density plots (**a**, **c**, **e**, and **g**) display the data distributions with raw data shown as dots. Unpaired mean (effect magnitude) is presented as circles or arrows with corresponding 95% confidence intervals (effect precision) presented as vertical lines. Negative and positive effects are presented as arrows pointing downwards and upward, respectively while neutral effects are presented as circles. Lighter hue indicates low concentration and darker hue indicates high concentration. Null models were used to predict the impacts of multiple-factor treatments on soil processes using individual treatment effects (**b**, **d**, **f**, and **h**). Error bars of multiple factor interactions in the null model plots were generated by boot-strapped values with 1000 iterations. Null models for low-concentration and high-concentration treatments are presented in the upper and lower panels, respectively. Factor levels are displayed in different colors:  - control;  - single-factor;  -three-factor; and  - four-factor treatments

We found that multiple-factor treatments negatively affected the β-glucosidase activity more strongly than the single-factor treatments (Fig. 2a). Remarkably, among the multiple-factor treatments, it was less affected in the no mask MP treatment (RAI). There were no distinct differences between actual data and null model assumptions (Fig. 2b). The single-factor treatment of each pharmaceutical compound showed neutral effects on phosphatase activity, while MP treatment significantly inhibited the enzyme activity (Fig. 2c). In the multiple-factor treatments, the phosphatase activity showed decreasing trends in the treatment with MP and high concentrations of pharmaceuticals (AIMP, ARMP, and RAIMP). Conversely, no MP treatment (RAI) at low concentration significantly increased phosphatase activity. We also found no significant differences in null model assumptions (Fig. 2D). In the case of both β-D-cellobiosidase (Fig. 2e) and N-acetyl-β-glucosaminidase (Fig. 2g) activities, high-concentration treatments showed an increasing trend in all treatments. In the single-factor treatment, both enzymes were significantly higher compared to the control group at high concentrations of R and A but significantly lower at low concentration of I. The multi-factor treatments showed the negative effects at low concentrations. This significant reduction was not observed when MP was excluded from the multi-factor treatments. In addition, there were significant differences between the actual data and the null model assumptions (Fig. 2f and h).

### Effects on soil microbial abundance

Bacterial and fungal abundance were not significantly affected by the treatments. Although bacterial abundance tended to be slightly lower under the treatment conditions compared to the control (Figure S2A), and fungal abundance had the opposite trend (Figure S2B), there was no change in the bacteria to fungi ratio (Figure S2C).

### Effects on soil physicochemical properties

Soil pH was significantly lower compared to the control group when pollutants were present (Figure S3A). The reduction was more pronounced in the single-factor treatments than in the three- or four-factor treatments. The actual data deviate significantly from the null model assumptions (Figure S3B). Conversely, there was no remarkable effect on water-stable aggregation under any treatment conditions (Figure S4B).

### Correlation among proxies for soil health and functions

**Fig. 3:**
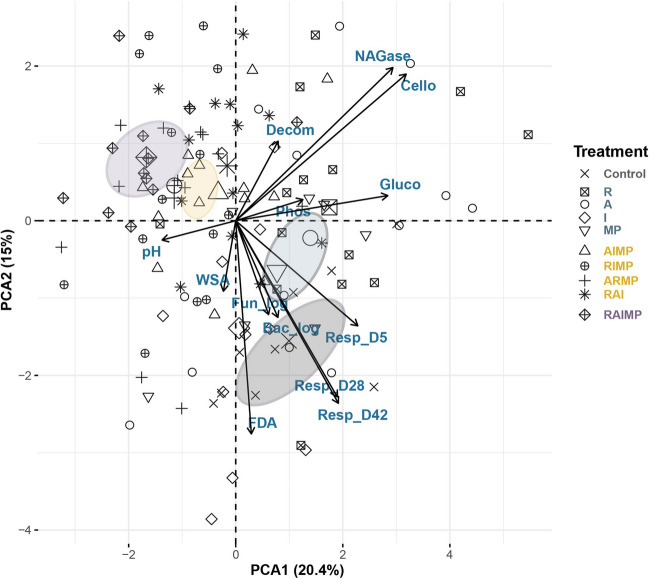
Principal component analysis (PCA) projecting the correlation between the tested soil parameters and the samples under the different treatment conditions. Samples under the different treatment conditions are distributed in the two-dimensional space represented by principal component axes 1 and 2 explaining 20.4 and 15% of variance, respectively. The soil parameters representing physico-chemical, enzymatic, and microbiological activities include soil pH, water-stable aggregates (WSA), β-glucosidase (gluco), β-D-cellobiosidase (cello), phosphatase (phos), N-acetyl-β-glucosaminidase (NAGase), FDA hydrolysis (FDA), litter decomposition (decom), soil respiration (resp_D5, resp_D28, resp_D42), and microbial abundance (bacteria and fungi). Arrows indicate direction and weight of variables. Colored dots represent different treatments regardless of concentration: control, remdesivir, R; azithromycin, A; ivermectin, I; mask microplastic, MP and their combinations (AIMP, RIMP, ARMP, RAI and RAIMP). Data distribution by factor level is indicated by concentration ellipses:  - control;  - single-factor;  -three-factor; and  - four-factor treatments

The general relationships among the different parameters and individual samples were evaluated using principal component analysis (Fig. 3). Microbial activities measured as soil respiration and FDA hydrolysis positively correlated with microbial abundance. Enzyme activities and litter decomposition, on the other hand, had a negative correlation with soil physical properties (i.e., pH and WSA). There was also a clear separation of treatment effects in the ordination space mirroring the combined responses of parameters assessed here.

## Discussion

Our study focuses on the individual and combined effects of COVID-19 pandemic–related drugs and microplastics derived from FFP2 masks on known proxies of soil health and functions. Results show that these pollutants can elicit changes in measured soil parameters when applied individually and in combination. Although there are many previous studies reporting the impacts of pharmaceutical drugs and mask microplastics in the environment, our study investigated the joint effects of COVID-19 pandemic–related drugs and mask MP on different soil processes and functions.

We investigated soil microbial activities by measuring soil respiration and FDA hydrolysis activity. Soil respiration measurements were taken at three different time points to compare the effects of short-term and long-term exposure. The substantial decline in soil respiration over time may be related to the general decrease in microbial activity due to substrate depletion (Hartley et al. 2008; Ölinger et al. 1996), toxicity of the pharmaceuticals, and the leachates from mask MP (Kim et al. 2020). We found a general trend across all time-point measurements, showing that inhibition was stronger under the multiple-factor treatments than the single-factor treatments. Null model testing of day 28 measurement indicated a synergistic interaction between pharmaceuticals and mask MP, resulting in more pronounced inhibition under AIMP, ARMP, and RIMP but not in the no MP treatments (RAI). Longer exposure (day 42) to the treatments further resulted in significant reductions both in single-factor treatments and multiple-factor treatments. These results support earlier findings on the negative effects of pharmaceutical products particularly antimicrobials (Butler et al. 2011; Cycoń et al. 2019; Girardi et al. 2011; W. Zhang et al. 2019) and microplastics (Lozano et al. 2021a; Zhao et al. 2021). Respiration data is further supported by the decrease in FDA hydrolysis, another known indicator of soil microbial activity, suggesting a potential reduction in microbial biomass (Schnürer and Rosswall 1982). However, in treatments of high concentrations of AIMP and low concentrations of ARMP, FDA hydrolysis was comparable to the control group. Null model testing indicates potential factor interaction, resulting in reduced negative effects compared to the individual components. This pattern was not obtained in other combinations; hence, it is difficult to make general predictions of the effects of multiple-factor treatments.

The addition of pharmaceuticals and microplastics to the soil may affect the microbial community and their activities (Lopez et al. 2021; Wu et al. 2021). In our study, there were no significant changes in the abundances of both bacteria and fungi. This did not conform with previous reports that antibiotic addition lowered bacterial abundance while increasing fungal abundance and biomass (Demoling et al. 2009; Tang et al. 2020). Antivirals were also found to cause changes in community structure with bacterial diversity being reduced (Slater et al. 2011). The neutral effects we obtained in this study may have been due to the concentrations used. Also, microbial biodegradation may have rendered these compounds and their metabolites non-toxic (Maldonado-Torres et al. 2018; Narayanan et al. 2023). Although bacterial abundance tended to be slightly lower in the treated soil than in the control and fungal abundance tended to be slightly higher, there is no evidence that the abundance ratio between these two groups have changed (Figure S2C). Similarly, litter decomposition rate was not significantly affected by the treatments, likely because fungal-to-bacterial ratio remains unaltered. Previous reports have linked the increase in soil fungal:bacterial ratio to increased litter decomposition, emphasizing the significant contribution of fungi to this important soil process (Malik et al.^2016^; M. Zhang et al. 2021). Despite the unaltered microbial abundance, the significant changes in metabolic activities suggest potential shifts in microbial community structure when exposed to these contaminants. Pharmaceutical products used in this study represent different classes (i.e., antiviral, antibacterial, and antiparasitic) with different mechanisms of action. Therefore, their presence in the soil samples can support certain functional groups while suppressing or inhibiting others (Izabel-Shen et al. 2022). Rillig et al. (2019) also found that microbial communities lost species, favoring stress-tolerant species when exposed to increasing numbers of global change factors. Microorganisms that are relatively more tolerant to the added antimicrobials may take advantage of the compounds as nutrient sources (Butler et al. 2011). For example, in our study, the addition of nitrogenous azithromycin and remdesivir had a positive effect on N-acetyl-β-glucosaminidase, an N-acquiring enzyme. β-D-cellobiosidase also appeared to be stimulated by high concentrations of the treatments whether applied as single or in combination. In both enzymes, high concentrations of pharmaceuticals with or without mask MP showed potential antagonistic interaction in the multiple-factor treatments as indicated by the null model testing, resulting in reduced negative effects. On the contrary, the treatments may be toxic to sensitive soil microbes (Cheng et al. 2021; Lagos et al. 2023; Rodríguez-González et al. 2023). The growth and metabolism of target species (e.g., antibiotics against bacteria) may have been suppressed by the addition of the compounds or by the toxic leachate from the mask MP resulting in lower enzymatic activities (Kim et al. 2020). The inhibitory effect of mask MP was evident in the β-glucosidase and phosphatase activities. In both enzymes, mask MP caused significant reduction when added as a single factor whereas this effect was not seen in the multiple-factor treatment where mask MP was excluded (i.e., RAI). This aligned with previous findings on the negative effects of microplastics on enzymatic activities due to their ability to change soil physicochemical properties (Yu et al. 2020; Zhao et al. 2021).

Single-factor treatments tend to lower soil pH possibly due to the innate pH of the compounds as exemplified by remdesivir, a highly acidic drug (Kumar et al. 2021). However, this negative effect tends to lessen under multiple-factor treatments. As biodegradation and biotransformation of the compounds take place, this can further alter soil pH (Carter et al. 2016). Consequently, soil pH modification may potentially affect soil processes, such as enzyme activities (Frankenberger and Johanson 1982). In our study, PCA results showed the inverse relationship between soil pH and enzymatic activities. N-acetyl-β-glucosaminidase and β-D-cellobiosidase, in particular, are stimulated at lower soil pH under the single-factor treatments. Conversely, the increase in soil pH in the multiple-factor treatments resulted in lower enzymatic activities. Soil pH also contributes to the sorption and persistence of pollutants in the system and how it will further impact soil health (Campillo-Cora et al. 2020; Chien et al. 2018; Franco et al. 2009; Kicińska et al. 2022; Y. Xu et al. 2021). While studies on microplastic effects on soil properties and functions have been building up in recent years, there is limited information on the effects of pharmaceutical products on soil aggregate formation and stability. Our study did not show any remarkable change in soil aggregation under treatment conditions. Considering the potential negative effects of pharmaceuticals on soil biota (L. Wang et al. 2019) and the significant contribution of the latter on soil aggregation (Lehmann et al. 2017), we see a need for further investigation, particularly on multiple-factor effects to bridge this gap.

## Conclusion

Our study uncovers the environmental effects of several materials connected to fighting the COVID-19 pandemic. We investigated the impacts of COVID-19 pharmaceutical drugs coupled with polypropylene microplastics from FFP2 masks on soil functions and properties. Given that pharmaceuticals and microplastics are continuously discharged into the environment, these products are ubiquitous and often occur as a complex mixture rather than isolated compounds. We found that pharmaceutical drugs and microplastics when applied individually can alter soil properties like pH, respiration, and important enzymes related to nutrient cycling. For some compounds, toxicity may not be clear when applied individually, but their combination may have substantial effects emphasizing the stronger effects of multiple factors in soil. Elevated concentrations of pharmaceuticals in soil such as in pandemic scenarios, may also lead to stronger interactions, either synergistically or antagonistically, between these compounds and other pollutants including microplastics. This underscores the importance of considering not only the direct impacts of pharmaceutical compounds but also their interactions with other pollutants when assessing environmental risks. Furthermore, this may aid policies geared towards One Health, recognizing the interdependence of human, animal, and environmental health. Recommendations may include enhancing waste treatment processes and establishing guidelines for the safe reuse of treated water and sludge in agriculture.

### Supplementary Information

Below is the link to the electronic supplementary material.

Supplementary file1 (DOCX 956 KB)

## Data Availability

All data used for analyses and plotting are available in https://doi.org/10.6084/m9.figshare.20474064.v1.
