# Supplementary material for: COVID-19 pandemic-related drugs and microplastics from mask fibers jointly affect soil functions and processes
Source: Environ Sci Pollut Res Int. 2024 Aug 5;31(38):50630–41. doi: 10.1007/s11356-024-34587-x (PMC11364614; doi:10.1007/s11356-024-34587-x)
Supplement: Supplementary file 1 — Supplementary file1 (DOCX 956 KB) [file 11356_2024_34587_MOESM1_ESM.docx]

**SUPPLEMENTARY MATERIAL**

**COVID-19 pandemic-related drugs and microplastics from mask fibers jointly affect soil functions and processes**

**Jeane A. dela Cruz^1.2^, Daniel Lammel^1.2^, Shin Woong Kim^1.2^, Mohan Bi^1.2^, Matthias C. Rillig^1.2^**

^1^ Institute of Biology, Freie Universität Berlin, 14195 Berlin, Germany

^2^ Berlin-Brandenburg Institute of Advanced Biodiversity Research, 14195 Berlin, Germany

Corresponding Author: **Matthias C. Rillig**

rillig@zedat.fu-berlin.de

tel. +49-30-838-53165

1. **Supplementary Methods**

The following response variables were measured: physico-chemical properties (pH and water-stable aggregates), microbial activity (soil respiration, FDA hydrolysis), nutrient cycling (litter decomposition rate and soil enzyme activities), and bacterial and fungal abundance.

**Soil respiration**. Soil respiration is used as a proxy for soil microbial activity. Lids of the falcon tubes were replaced with modified lids fitted with a rubber septum to facilitate sampling of CO_2_. Tubes were flushed with CO_2_-free air for 5 minutes.  To ensure that flushing was sufficient, 1 ml of gas was sampled from the headspace using a medical syringe prior to incubation for 2 hours. After incubation, another 1 ml of air was sampled and injected into an infrared gas analyzer (LiCOR 6400xt, Lincoln, NE, USA) to measure CO2 concentration. After measurement, the original vented lids were replaced.

**pH Measurement**. 5 g of soil was mixed with 12.5 ml 0.01 M CaCl_2_ solution in a 50 ml falcon tube. Tubes were mixed quickly with a vortex and centrifuged for 10 min at 4600 rpm. The supernatant was used to measure the pH using a pH meter (Knick, Germany).

**Enzyme assays**. Soil enzyme activities relating to nutrient cycling were measured such as β-glucosidase (cellulose degradation), β-D-cellobiosidase (cellulose degradation), N-acetyl-β-glucosaminidase (chitin degradation), and phosphatase (organic phosphorus mineralization). Soil samples were stored at 4 °C and enzyme assays were done within 2 weeks using high throughput microplate assay. First, p-nitrophenyl (pNP)-linked substrate solutions were prepared separately for each assay: 5 mM pNP-β-D-glucopyranoside (Sigma-Aldrich, MO, USA, Sigma no. N7006), 2 mM pNP-β-D-cellobioside (Sigma no. N5759), 2 mM pNP-N-acetyl β-D-glucosaminide (Sigma no. N9376) and 5 mM pNP- phosphate disodium salt hexahydrate (Sigma no. 71768). Soil slurry was prepared by mixing about 5 g of soil sample with 8 ml of 50 mM acetate buffer. 150 µl of the slurry were immediately pipetted into 6 wells of a 96-well microplate vortexing in between to ensure that soil particles were well-suspended. Four of these wells were added with 150 µl of the freshly prepared substrate solution and the remaining two wells were added with 150 µl acetate buffer as control. The microplates for phosphatase and β-glucosidase were then incubated for 2 hrs and NAGase and cellobiosidase were incubated for 4 hrs. After incubation, microplates were centrifuged at 3000 rpm for 5 min. 100 µl supernatant from each sample was carefully withdrawn and transferred to fresh microplates and added with 200 µl 0.05 M NaOH to stop the reaction. Using a microplate reader (BioRad, Benchmark Plus, Japan), absorbance was measured spectrophotometrically at 410 nm. Using a standard curve, measured absorbances were converted to pNP concentration (μmol per gram of dry soil per hour).

**Fluorescein diacetate hydrolase** (FDA) hydrolysis was used as an indicator of general microbial activity using high throughput microplate assay. Soil slurry was prepared by mixing 1 g of soil with 9 ml phosphate buffer. The mixture was mixed thoroughly with a vortex and 270 µl was pipetted on 6 wells. 40 µl of fluorescein diacetate (FDA) solution was added to 4 of the 6 wells while the remaining 2 wells served as control containing only phosphate buffers and soil slurry. The microplates were incubated for 1 h at 30 °C. After incubation, plates were centrifuged for 5 min at 3000 rpm. 200 µl supernatant was carefully pipetted out and transferred to a new microplate and the extracted fluorescein was measured at 490 nm using a microplate reader (BioRad, Benchmark Plus, Japan). A standard curve was used to convert measured absorbance to hydrolyzed FDA (mg h^-1^ g^-1^ dry soil).

**Water-stable soil aggregates**. The stability of soil aggregates (>250 µm) against a disintegrating force, in this case, water was measured using an established protocol (Kemper and Rosenau, 1986; Liang et al., 2019). 4.0 g dried soil from each sample was placed on a sieve with a mesh size of 0.25 mm and placed in a pre-weighed weighing boat. Weighing boats were carefully filled with deionized water and via capillary re-wetting allowed soil to soak for 5 minutes. After which, sieves containing the soil samples were inserted into the sieving machine (Agrisearch Equipment, Eijkelkamp, Giesbeek, Netherlands) and allowed to tumble in water for 3 min. The fraction left on the sieves was collected to the corresponding weighing boats and oven-dried for 24h at 60°C and aggregates were weighed (dry matter).  To extract the sand and other organic debris, the dried matter was crushed using the same sieve and washed with deionized water until the water running through was clean. All the remaining fragments in the sieve were again collected on the weighing boat and dried overnight at 60°C. Final weight (coarse matter) was obtained and %WSA was calculated using the formula:

%WSA = [(dry matter – coarse matter) / (4 g – coarse matter)] * 100

**Decomposition**. The rate of organic matter decomposition by soil microbes was measured using litter bag. Approximately 300 mg of green tea leaves (Lipton Green Tea Sencha, Japan) were used to fill prepared nylon bags of (2.5 x 1.5 cm) dimension and sealed using an impulse sealer (Mercier Corp). Litter bags were briefly microwaved for 30 seconds to minimize contamination. The bags were inserted in the middle depth of the soil of the microcosm and collected at the end of the incubation. To remove adhering soil particles, litter bags were rinsed with deionized water and subsequently oven-dried at 60°C. The reduction in mass was calculated and was used as an indication of decomposition rate (%).

**Soil DNA Extraction and standard preparation**. Approximately 250 mg of well-mixed soil is harvested at the end of the incubation and stored at -20°C. Following the manufacturer’s instruction, soil DNA was extracted using DNeasy PowerSoil Pro Kit (QIAGEN GmbH, Germany). Extracted DNA are kept at -20°C until used for quantitative PCR (qPCR). 1 μl of DNA extracted from each sample were pooled and amplified using Biometra Tone thermocycler (Analytik Jena, Jena, Germany). Amplification was then examined on 1.0% agarose gel using electrophoresis (expected amplicon size is 350 bp). Following manufacturer’s instructions, the PCR product was subsequently purified using PCR magnetic beads (NucleoMag NGS Clean-up and Size Selection, MACHEREY-NAGEL GmbH & Co.KG, Düren, Germany) in 0.8:1 ratio (bead:PCR product). The DNA concentration of the PCR products was measured using Qubit 3 Fluorometer (Fisher Scientific GmbH, Germany). Afterwhich, calibration standards were prepared by initially diluting the PCR product to 1x10^9^ copies μl^-1^ and further diluted to 1x10^2^ copies μl^-1^ by serial dilution.

***Bacterial abundance***. Quantitative PCR (qPCR) targeting the 16S rRNA gene was used to estimate bacterial abundance. Soil DNA was amplified using universal primers 515F (5′‐GTGCCAGCMGCCGCGGTAA‐3′) and 806R (5’‐GGACTACHVGGGTWTCTAAT‐3’) for bacteria. A 20 μl reaction mix was prepared: 4.0 μl of 5x KAPA HiFi Fid buffer with Mg, 0.4 μl of 10 mM KAPA dNTP Mix, 0.5 μl of 10 μM of each primer, 0.2 μl of 1 U μl-1 153 of 154 KAPA HiFi polymerase (Kapa Biosystems, Woburn, MA, USA), 13.4 μl PCR-grade water and 1 μl of DNA template.  Using the thermocycler CFX 96 Real‐Time System (Touch 1009, Bio‐Rad Lab., Inc, USA), qPCR conditions were as follows: a denaturation step for 3 min at 95°C, 40 cycles of denaturation for 15 s at 95°C, annealing for 30 s at 52°C, and elongation for 30 s at 68°C, and a final elongation step of 5 min at 68°C. All analyses were performed in duplicates.  DNA standards and the negative controls using PCR-grade water were also included in each 96-well plate. Obtained copy numbers (log transformed) were used to indicate bacterial abundance.

***Fungal abundance.*** Quantitative PCR (qPCR) targeting the fungal ITS region was used to estimate fungal abundance. qPCR for fungi was performed using primers ITS7F and ITS4R using the thermocycler CFX 96 Real‐Time System (Touch 1009, Bio‐Rad Lab., Inc, USA). A 20 μl reaction mix was prepared: 4.0 μl of 5x KAPA HiFi Fid buffer with Mg, 0.4 μl of 10 mM KAPA dNTP Mix, 0.5 μl of 10 μM of each primer, 0.2 μl of 1 U μl-1 153 of 154 KAPA HiFi polymerase (Kapa Biosystems, Woburn, MA, USA), 13.4 μl PCR-grade water and 1 μl of DNA template.  Using CFX 96 Real‐Time System (Touch 1009, Bio‐Rad Lab., Inc, USA), qPCR cycle was as follows: a denaturation step for 3 min at 95°C, 30 cycles of denaturation for 20 s at 98°C, annealing for 30 s at 53°C, and elongation for 30 s at 72°C, and a final elongation step of 5 min at 72°C. All analyses were performed in duplicates.  DNA standards and the negative controls using PCR-grade water were also included in each 96-well plate. Obtained copy numbers (log transformed) were used to indicate fungal abundance.

1. **Supplementary Tables**

**Table S1**. Experimental Design

| **Combination Level** | **Factors (Pollutants)^a^** | **Concentration^b^** | **Replicates per concentration** |
| --- | --- | --- | --- |
| 0  (Control) | none | none | 10 |
| 1-factor | R  A  I  MP | High/Low  High/Low  High/Low  0.04% | 8  8  8  8 |
| 3-factor | A-I-MP  R-I-MP  A-R-MP  R-A-I | High/Low  High/Low  High/Low  High/Low | 8  8  8  8 |
| 4-factor | R-A-I-MP | High/Low | 8 |

^a^R= Remdesivir, A== Azithromycin, I= Ivermectin, MP= Microplastics

**^b^**low and high concentrations are presented in Table S2

**Table S2**. Basic information of the pharmaceutical compounds used.

| **Compound** | **Manufacturer** | **CAS registry no.** | **Class** | **Chemical Structure** | **Chemical formula** | **Molecular weight**  **(g/mol)** | **pKa** | **Mechanism of Action** | **Working concentration**  **(mg/kg)** | |
| --- | --- | --- | --- | --- | --- | --- | --- | --- | --- | --- |
|  |  |  |  |  |  |  |  |  | **Low**^a^ | **High**^b^ |
| Remdesivir | Cayman Chemical | 1809249-37-3 | Antiviral (necluoside analogue, broad-spectru) | 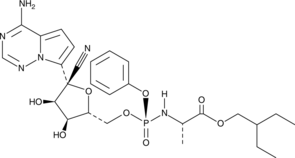 | C_27_H_35_N_6_O_8_P | 602.585 | 10.23 | Induces RNA chain termination and inhibits viral polymerases | 0.011 | 1.1 |
| Azithromycin | Sigma-Aldrich | 83905-01-5 | Macrolide antibiotic (broad-spectrum) | 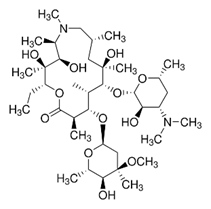 | C_38_H_72_N_2_O_12_ | 748.98 | 8.5 | Inhibition of protein synthesis | 0.014 | 1.4 |
| Ivermectin | Sigma-Aldrich | 70288-86-7 | Antiparasitic | 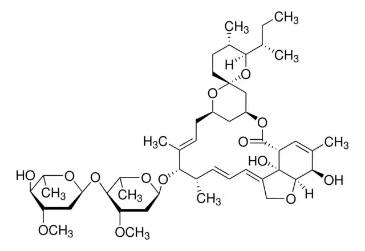 | C_95_H_146_O_28_ | 874.7 | 6.5 | Increased permeability of cell membrane to chloride ions | 0.05 | 5 |

^a^ based on pre-pandemic Maximum Environmental Concentration (MEC) or Maximum Reported Concentration (MRC)

^b^ 100 times higher than low concentration to exemplify substantial increase such as a pandemic scenario

1. **Supplementary Figures**


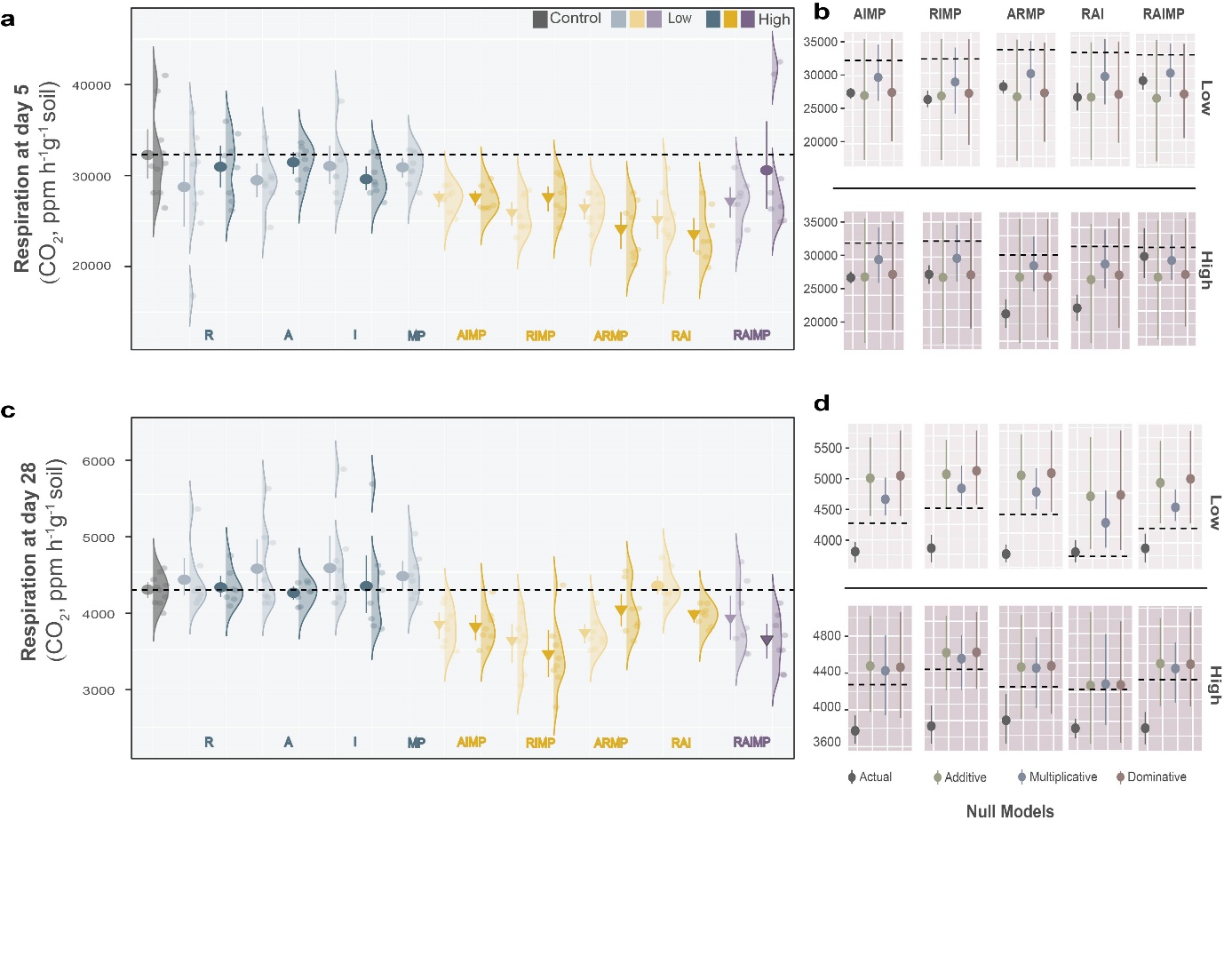


**Fig. S1**  Effects of the individual treatments (remdesivir, R;  azithromycin, A; ivermectin, I; mask microplastic, MP) and combinations of pharmaceutical drugs and microplastics on soil respiration measured on day 5(A) and day 28 (B). Density plots (A, C) display the data distributions with raw data shown as dots. Unpaired mean (effect magnitude) is presented as circles or arrows with corresponding 95% confidence intervals (effect precision) presented as vertical lines. Negative and positive effects are presented as arrows pointing downwards and upward, respectively while neutral effects are presented as circles. Lighter hue indicates low concentration and brighter hue indicates high concentration. Null models were used to predict the impacts of multiple-factor treatments on soil processes using individual treatment effects (B, D). Error bars of multiple factor interactions in the null model plots were generated by boot-strapped values with 1000 iterations. Null models for low-concentration and high-concentration treatments are presented in the upper and lower panels, respectively. Factor levels are displayed in different colors:
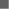
- control;
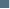
- single-factor; 
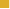
-three-factor; and
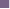
- four-factor treatments


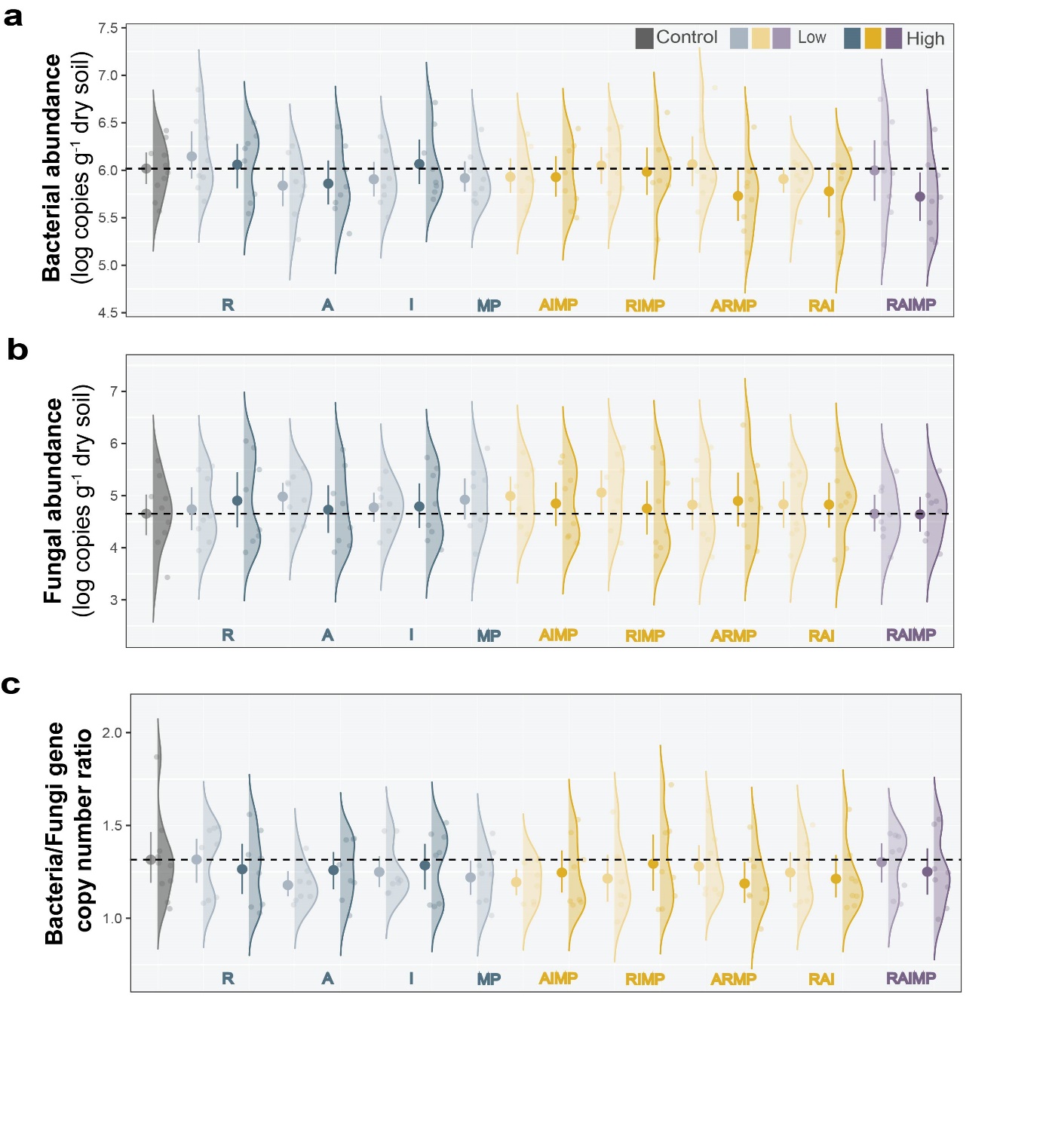


**Fig. S2**  Effects of the individual treatments (remdesivir, R;  azithromycin, A; ivermectin, I; mask microplastic, MP) and combinations of pharmaceutical drugs and microplastics on microbial abundance. Density plots display the data distributions with raw data shown as dots. Unpaired mean (effect magnitude) is presented as circles with corresponding 95% confidence intervals (effect precision) presented as vertical lines. Factor levels are displayed in different colors:
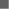
- control;
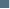
- single-factor; 
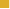
-three-factor; and
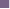
- four-factor treatments. Lighter hue indicates low concentration and hue shade indicates high concentration


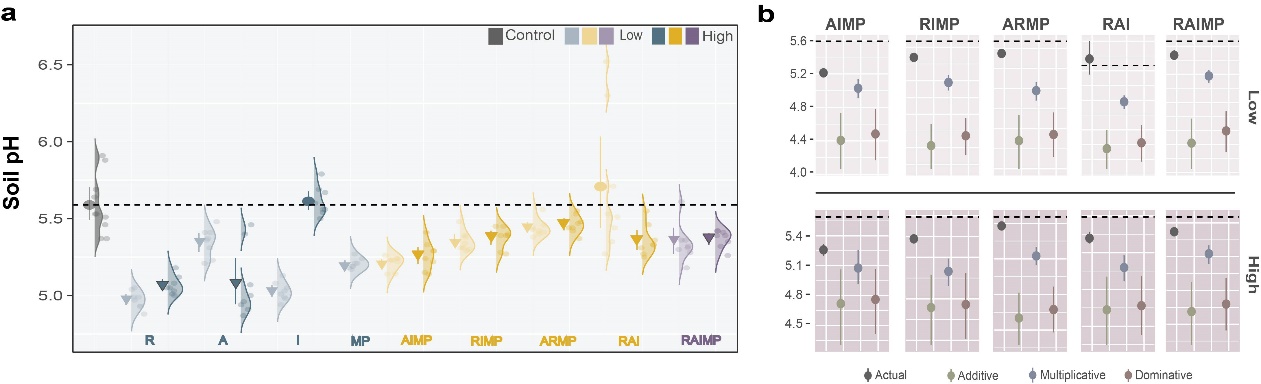


**Fig. S3**  Effects of the individual treatments (remdesivir, R;  azithromycin, A; ivermectin, I; mask microplastic, MP) and combinations of pharmaceutical drugs and microplastics on soil pH. Density plot (A) displays the data distributions with raw data shown as dots. Unpaired mean (effect magnitude) is presented as circles or arrows with corresponding 95% confidence intervals (effect precision) presented as vertical lines. Negative and positive effects are presented as arrows pointing downwards and upward, respectively while neutral effects are presented as circles.  Lighter hue indicates low concentration and brighter hue indicates high concentration. Null models were used to predict the impacts of multiple-factor treatments on soil pH using individual treatment effects (B). Error bars of multiple factor interactions in the null model plots were generated by boot-strapped values with 1000 iterations. Null models for low-concentration and high-concentration treatments are presented in the upper and lower panels, respectively. Factor levels are displayed in different colors:
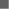
- control;
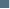
- single-factor; 
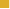
-three-factor; and
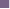
- four-factor treatments


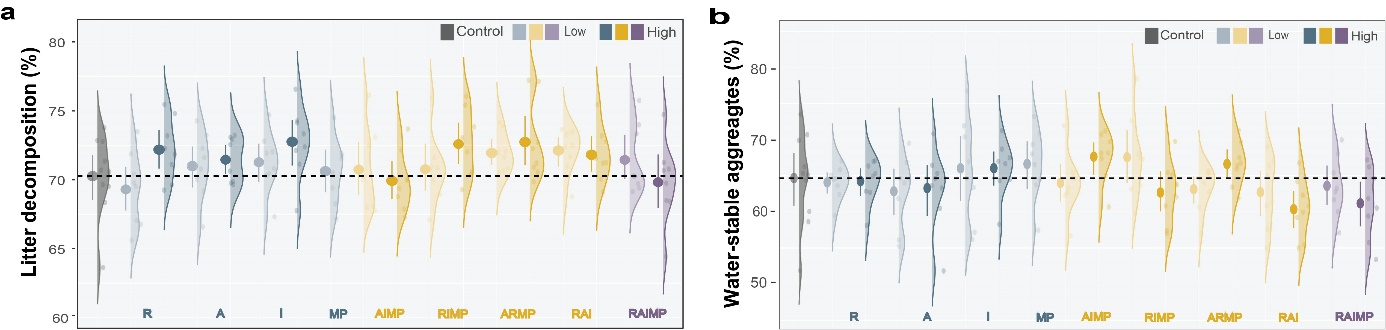


**Fig. S4**  Effects of the individual treatments (remdesivir, R;  azithromycin, A; ivermectin, I; mask microplastic, MP) and combinations of pharmaceutical drugs and microplastics on litter decomposition (A) and water-stable aggregates (B). Density plot displays the data distributions with raw data shown as dots. Unpaired mean (effect magnitude) is presented as circles or arrows with corresponding 95% confidence intervals (effect precision) presented as vertical lines. Lighter hue indicates low concentration and brighter hue indicates high concentration. Factor levels are displayed in different colors:
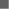
- control;
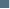
- single-factor; 
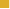
-three-factor; and
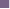
- four-factor treatments
